# Predicting visual acuity of treated ocular trauma based on pattern visual evoked potentials by machine learning models

**DOI:** 10.3389/fcell.2025.1619956

**Published:** 2025-08-01

**Authors:** Hongxia Hao, Jiemin Chen, Yifei Yan, Qi Zhang, Zhilu Zhou, Wentao Xia

**Affiliations:** ^1^ Shanghai Key Laboratory of Forensic Medicine, Shanghai Forensic Service Platform, Academy of Forensic Science, Shanghai, China; ^2^ The SMART (Smart Medicine and AI-based Radiology Technology) Lab, Shanghai Institute for Advanced Communication and Data Science, Shanghai University, Shanghai, China; ^3^ School of Communication and Information Engineering, Shanghai University, Shanghai, China; ^4^ Department of Forensic Medicine, Guizhou Medical University, Guizhou, Guiyang, China

**Keywords:** visual evoked potential, visual acuity, best corrected visual acuity, machine learning, pattern visual evoked potentials

## Abstract

**Purpose:**

To develop effective machine learning models that analyze pattern visual evoked potentials (PVEPs) to predict the stabilized visual acuity (VA) of patients with treated ocular trauma.

**Methods:**

This experiment included 260 patients (220 males, average age 42.54 years) with unilateral ocular trauma. Four different machine learning algorithms, namely, support vector regression (SVR), Bayesian ridge (BYR), random forest regression (RFG), and extreme gradient boosting (XGBoost), were used to predict best corrected visual acuity (BCVA) values. Various ophthalmic parameters were input into the above algorithms for model training, and the performance of the algorithms was analyzed from the difference between the prediction value and the ground truth. Among the BCVA measured at least 6 months after injury was set as the ground truth. The best-performing model was further developed by tuning different parameter combinations.

**Results:**

All models achieved high diagnostic performance, with accuracy values ranging from 0.7875 to 0.8133. The XGBoost model predicted BCVA values with the lowest mean absolute error (MAE), at 0.1598 logarithm of the minimum angle of resolution (logMAR); the lowest root mean square error (RMSE), at 0.2402 logMAR; and the highest accuracy, at 0.8959.

**Conclusion:**

Promising outcomes in BCVA prediction were achieved by the PVEP-trained machine learning models, which will be helpful in the clinical evaluation of patients after ocular trauma.

## 1 Introduction

Medical diagnosis plays a vital role in real-life clinical practice and is extremely challenging. Artificial intelligence (AI) has gradually risen to prominence and made revolutionary progress in the medical field in recent years. With the help of AI, predictive systems are assisted in solving some disease-related problems and predicting clinical outcomes to a certain extent. And Ophthalmology is an essential branch of medicine. In the field of Ophthalmology, AI technology can be applied to promote the development of disciplines, bring technological revolution, and establish new medical diagnosis systems. The diagnosis of diseases and the prediction of vision outcomes are performed essentially for treatment, recovery and the choice of surgical plans for ophthalmic patients. Machine learning (ML), deep learning (DL), and other AI technologies combined with various ophthalmic examination methods have been proved to play an essential role in the clinical field (Ting et al., 2019; Bali and Bali, 2021). According to ophthalmic examination data, disease types and related features are automatically recognized and judged by AI.

At present, the application of AI in medical treatment is mainly focused on image-based diagnostic technology, and numerous studies have compellingly demonstrated the application potential of AI in disease diagnosis. In terms of ophthalmic diagnosis, AI is gradually recognized for identifying diseases, classifying test results and predicting outcomes. The application of AI in ophthalmology is relevant to image enhancement (Benet and Pellicer-Valero, 2022) and the diagnosis of ophthalmic diseases such as age-related macular degeneration (AMD) (Dow et al., 2022), diabetic retinopathy (DR), diabetic macular edema, and retinopathy of prematurity (ROP) as well as the prediction of outcomes following cataract treatment (Gutierrez et al., 2022). Currently, AI is used by most ophthalmologists to help analyze optical coherence tomography (OCT) images, fundus photographs, fundus fluorescence angiography images, B-ultrasound images, and other medical imaging data in order to inform their decision-making. To characterize the state of research on these applications, Boudry collected and analyzed 1,356 articles from the past 53 years and found that retina and glaucoma are the main topics to which AI has been applied in ophthalmological research and studies have mainly focused on computer methods and other related technical research (Boudry et al., 2022). Borra et al. devoted using Google’s new AI technology to automatically identify and extract retinal vascular networks from color retinal images and then evaluated retinal vascular caliber to predict cardiovascular disease (Borra et al., 2022).

Eye trauma is more common in various traffic accidents and personal injuries. Vision is a factor of concern in eye trauma, which often involves relevant compensation, clinical treatment effects, and providing a basis for the trial of cases. PVEP is an objective visual detection method not subject to subjective conscious images. The P-VEP response in the brain’s visual cortex was induced by using a black-and-white flipped checkerboard image, mainly by observing the differentiation of waveforms and the amplitude and peak of the P100 component, compared with normal reference values or normal laboratory values. Based on binocular comparison, the results of the P-VEP test were analyzed to evaluate the visual acuity. The research focus of this paper is to predict the healing visual acuity of patients with ocular trauma to provide a reference for the forensic clinical visual acuity assessment of visual impairment after ocular trauma. The research results will be helpful for the follow-up forensic and healing assessments. The main purpose of this study is to analyze individual ophthalmic examination data such as pattern visual evoked potentials (PVEPs) using a variety of algorithms to evaluate best corrected visual acuity (BCVA) values and to identify the optimal algorithm and optimal parameter combination.

## 2 Methods

### 2.1 Participants

This article is a retrospective cohort study. A dataset including 260 patients with ocular trauma was drawn from the database of the Department of Forensic Clinical Medicine between 2008 and 2021 at the Academy of Forensic Science (Shanghai, China) according to a set of eligibility criteria that will be detailed below, and an additional 20 validation sets were collected. These data comprised 220 males (84.6%) and 40 females (15.4%), with an average age of 42.54 years. The demographics of all patents are summarized in Table 1. The inclusion criteria were as follows: (1) monocular trauma, (2) monocular visual dysfunction, and (3) BCVA >1.3 logarithm of the minimum angle of resolution (logMAR) of the injured eye. The exclusion criteria were situations with (1) patient-reported diseases that could lead to visual dysfunction, such as AMD, diabetes, or cataracts; (2) a previous history of eye trauma before the injury for which the patients were being treated at the time; (3) binocular visual impairment after an eye injury; and (4) visual acuity (VA) that had not yet stabilized as a result of ongoing recovery from the injury. The subjects selected for this study had complete ophthalmic history data, and the VA tested during identification was matched with the basis of injury. Pseudo-blind patients who exaggerated the degree of their visual impairment were excluded. The details of the patients included in this study are shown in Table 1.

**TABLE 1:** Patient characteristics.

Demographics	Data set	Validation set
Country	China	China
Number of eyes	260	20
Age (mean ± SD, years)	42.54 ± 14.03	45.7 ± 16.55
Ethnicity	East-south Chinese	East-south Chinese
Male gender (%)	220 (84.6%)	18 (90.0%)
Number of poor VA in each BCVA range		
<0.50 logMAR	173	6
≥0.50 logMAR	87	14

VA, visual acuity; BCVA, best corrected visual acuity; logMAR, logarithm of the minimum angle of resolution.

The types of trauma in this article include relatively minor trauma and conditions that seriously affect healed vision, such as ocular appendage injury, corneal contusion and superficial scratch, iris-ciliary injury, corneal laceration, corneal limbal rupture, scleral rupture, anterior chamber hemorrhage, traumatic cataract affecting the central visual axis, lens dislocation, vitreous hemorrhage, little or limited detachment or absence of pigment membrane, open globe injury with prolapse of ocular contents, intraocular foreign body injury, complicated by traumatic glaucoma, macular holes, concurrent intraocular infections, optic nerve atrophy, and other conditions.

### 2.2 Variables

The experimental PVEP data were obtained by Roland Consult (Electrophysiological Diagnostic Systems, Brandenburg, Germany) and LKC Technologies (UTAS visual electrodiagnostic test system, Gaithersburg, MD, United States). PVEP is done at least 6 months after the eye injury has occurred and the injury has stabilized. The examination process was carried out following the guidelines on visual electrophysiological examination for clinical forensic medicine. During the PVEP test, if it is found that the gaze of the inspected eye is poor, there is abnormal interference during the test, or the waveform is difficult to judge, the test will be repeated to confirm the reliability of the waveform. We constructed the program with the following variables: the amplitude and peak time (PT) of P100 for 15′and 60′checker size, the patient’s uncorrected visual acuity, the refractive status of each eye (spherical, cylindrical power and cylindrical axis), the BCVA values of the injured and healthy eyes, the patient’s previous ophthalmic history, and the Ocular Trauma Score (OTS) category. The mean spherical equivalent (SE) was calculated as follows: mean SE = sphere + (cylinder/2). We assessed the performance of the injured eye using the amplitude ratio and PT delay rate, calculated as follows:

Amplitude ratio = injured eye amplitude/healthy eye amplitude, PT delay rate = (injured eye PT - healthy eye PT)/healthy eye PT.

### 2.3 Algorithms

**FIGURE 1:**
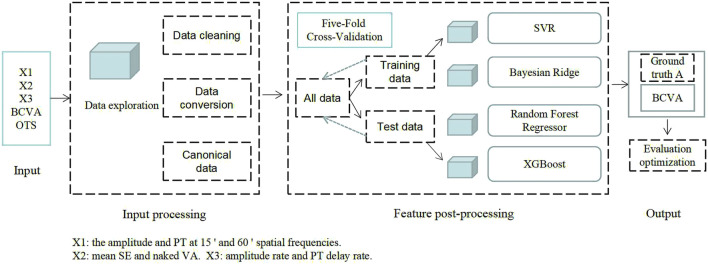
General pipeline of multiple machine learning algorithms.

To predict BCVA outcomes in patients with ocular trauma, we applied the support vector regression (SVR), Bayesian ridge (BYR), random forest regression (RFG) and extreme gradient boost (XGBoost) algorithms to predict BCVA. Figure 1 shows the flow diagrams for the machine learning-based task and the model outputs are BCVA.

**FIGURE 2:**
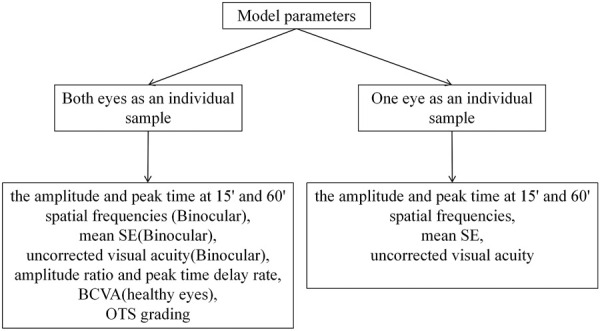
Feature input details.

The process of machine learning model analysis based on electrophysiological features consisted mainly of the following steps. (1) Feature extraction (as described in the variables section above). (2) Feature selection. The specific process: All features (Figure 2) are used as model inputs to predict BCVA, and then Shapley additive explanation (SHAP) is used to calculate and rank the Shapley Value of all features corresponding to the model. All features are taken as inputs, and the filtered features are used as group A using the Lasso method. All the data were divided into two groups with logMAR 0.5 as the cut-off value. The independent sample t-test was performed on all the features of the two data groups. The features with significant differences were selected as group B. The intersection of groups A and B is taken, and the features with significant Shapley value are added as the final feature combination. (3) Model prediction. All feature groups were first standardized, and then four different machine learning models were used to complete the BCVA prediction. (4) Model evaluation. Different evaluation metrics were used to measure the merits of the models for the BCVA prediction.

XGBoost is an integrated model that combines multiple weak learners into a strong learner; this model has been widely used in various competitions and has achieved good results owing to its outstanding efficiency and high prediction accuracy. Similar to other algorithms based on integration learning, XGBoost builds a loss function and forms a strong learner by generating multiple weak learners iterating progressively forward. In addition, the structure of the loss function is optimized by adding a regularization term, which can significantly improve the robustness of the model and reduce the risk of overfitting.

### 2.4 Evaluation

For the dataset, we used a fivefold cross-validation method to divide the training and test sets separately. The Pearson correlation coefficient (PCC) was used to assess the relationship between the ground truth and the predicted BCVA. The root mean square error (RMSE) and the mean absolute error (MAE) were used to provide a quality measure for our predictions of BCVA, which shows how close they are to the ground truth. Their definitions are shown in Equations 1–3.

$$\text{RMSE}=\sqrt{\frac{\sum_{i=1}^{\;N}{\left({\tilde{y}}_{i}-y_{i}\right)}^{2}}{N}}$$ (1)

$$\text{MAE}=\frac{\sum_{i=1}^{\;N}\left|{\tilde{y}}_{i}-y_{i}\right|}{N}$$ (2)

$$\text{PCC}=\frac{\sum_{i=1}^{\;N}\left({\tilde{y}}_{i}-\frac{1}{N}\sum_{i=1}^{\;N}{\tilde{y}}_{i}\right)\left(y_{i}-\frac{1}{N}\sum_{i=1}^{\;N}y_{i}\right)}{\sqrt{\sum_{i=1}^{\;N}{\left({\tilde{y}}_{i}-\frac{1}{N}\sum_{i=1}^{\;N}{\tilde{y}}_{i}\right)}^{2}}\sqrt{\sum_{i=1}^{\;N}{\left(y_{i}-\frac{1}{N}\sum_{i=1}^{\;N}y_{i}\right)}^{2}}}$$ (3)

where ${\tilde{y}}_{i}$ indicates the actual BCVA, $y_{i}$ indicates the predicted BCVA and N indicates the number of input samples.

Continuous variables are described as the mean ± standard deviation. All computations were performed in Python and SPSS (IBM Chicago, United States) using a Dell computer with an Intel (R) Core (TM) i7-10870H CPU @ 2.20 GHz and 32 GB RAM.

## 3 Results

This work validates the performance of the models and confirms their generalizability by using a fivefold cross-validation approach. As shown in Table 2, the fivefold validation showed stable and promising results for the four algorithms in predicting BCVA. Notably, the XGBoost algorithm showed the highest PCC of 0.8133, the lowest MAE of 0.2148 (±0.02) and the lowest RMSE of 0.2751 (±0.02) in the fivefold cross-validation. As the best performer, the XGBoost algorithm was selected for evaluation and development.

**TABLE 2:** Results of four algorithms in predicting BCVA with fivefold cross-validation.

Algorithms	PCC	MAE	RMSE
SVR	0.7875 ± 0.03	0.2364 ± 0.01	0.2929 ± 0.01
BYR	0.7741 ± 0.04	0.2386 ± 0.02	0.2980 ± 0.01
RFG	0.7960 ± 0.05	0.2222 ± 0.02	0.2869 ± 0.02
XGBoost	0.8133 ± 0.02	0.2148 ± 0.02	0.2751 ± 0.02

PCC, Pearson correlation coefficient; MAE, Mean absolute error; RMSE, Root mean square error.

**FIGURE 3:**
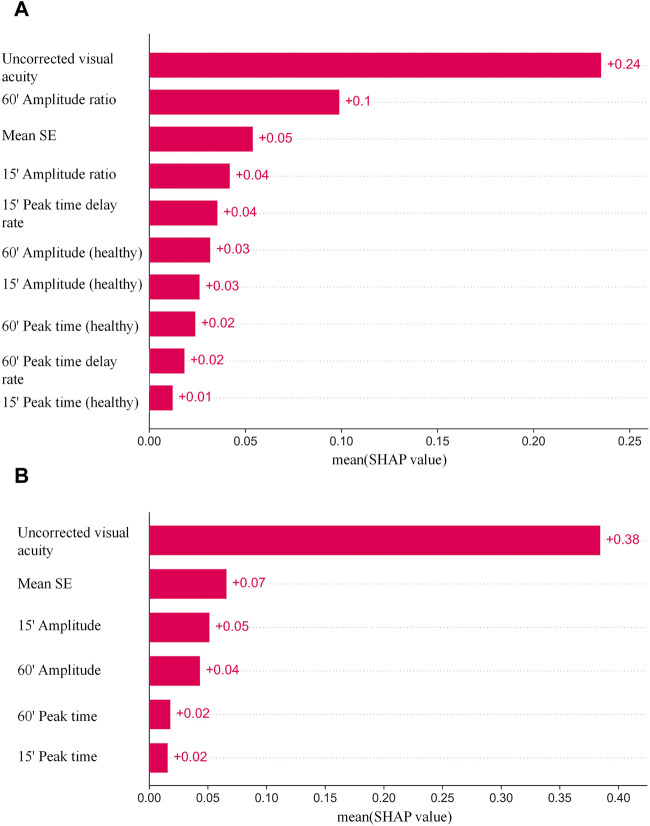
**(A)** The SAHP of both eyes as an individual sample. **(B)** The SAHP of one eye as an individual sample.

The SHAP was used to visualize the importance of features with optimal hyperparameters for the XGBoost model with the best results. The SAHP of both eyes as an individual sample is shown in Figure 3A, and one eye as an individual sample is shown in Figure 3B. In the Figure above, the uncorrected visual acuity strongly supports visual acuity prediction, as does peak time weakly support visual acuity prediction.

Different combinations of features were chosen as inputs for the XGBoost model, representing two main categories: one approach is to take the injured eye and healthy eye of the same subject as a whole, and the second is to take the characteristics of a single eye as an individual. The parameters included in the experiment were amplitude ratio, PT delay rate, amplitude and PT at 15′and 60′spatial frequencies, BCVA, mean SE, uncorrected visual acuity and OTS category. Models with different combinations of parameters are established using XGBoost, and the results are shown in Table 3. It can be seen that in the classification of both eyes as an individual sample, the best learner in all cases was the model which inputs used in amplitude ratio, PT delay rate, mean SE and the uncorrected visual acuity of injured eyes, the amplitude and PT of healthy eyes at 15′and 60′spatial frequencies. The model showed the highest PCC (0.8133 [±0.05]), the lowest MAE (0.2148 logMAR [±0.02]), and the lowest RMSE (0.2751 logMAR [±0.02]) in the fivefold cross-validation. In the classification of BCVA prediction with monocular as an individual, mean SE, uncorrected visual acuity, amplitude and PT for 15′and 60′of all eyes were included in the model. The predictive model exhibited stable results with PCC, MAE and RMSE values of 0.8959 (±0.02), 0.1598 (±0.01) and 0.2402 (±0.02), respectively, in the fivefold cross-validation.

**TABLE 3:** Results of XGBoost models with different combinations of features in predicting BCVA. The best indices are denoted by bold font.

Model parameters	N	PCC	MAE	RMSE
Both eyes as an individual sample				
X1(Binocular), good BCVA, poor X2, OTS grading	221	0.7943 ± 0.09	0.2295 ± 0.02	0.284 ± 0.03
X1 and X2 (Binocular), good BCVA, OTS grading	221	0.7891 ± 0.05	0.2252 ± 0.02	0.2847 ± 0.03
Poor X1, X2 (Binocular) and X3	249	0.8111 ± 0.05	0.2143 ± 0.03	0.2752 ± 0.03
Good X1, poor X2 and X3	249	**0.8133** ± **0.02**	**0.2148** ± **0.02**	**0.2751** ± **0.02**
Good X1 and BCVA, poor X2, and X3	249	0.8011 ± 0.04	0.2279 ± 0.02	0.2913 ± 0.02
Good X1, good BCVA, X2 (binocular) and X3	249	0.7897 ± 0.05	0.2289 ± 0.02	0.2859 ± 0.03
X2 (binocular), X3, and OTS grading	249	0.7808 ± 0.08	0.217 ± 0.02	0.2856 ± 0.02
Good X1, X2 (binocular), X3, and OTS grading	249	0.8007 ± 0.04	0.2169 ± 0.02	0.2815 ± 0.03
One eye as an individual sample				
Poor X1 and X2	252	0.7975 ± 0.04	0.223 ± 0.02	0.2822 ± 0.02
X1 and X2	504	**0.8959** ± **0.02**	**0.1598** ± **0.01**	**0.2402** ± **0.02**
X1 and X2 (Only poor data were tested)	504	0.7932 ± 0.03	0.2424 ± 0.02	0.3075 ± 0.02

X1, The amplitude and peak time at 15′ and 60′spatial frequencies; X2, mean SE and uncorrected visual acuity; X3, Amplitude ratio and peak time delay rate; BCVA, Best corrected visual acuity; OTS, Ocular trauma score; PCC, Pearson correlation coefficient; MAE, Mean absolute error; RMSE, Root mean square error; Good, healthy eyes; Poor, injured eyes.

**FIGURE 4:**
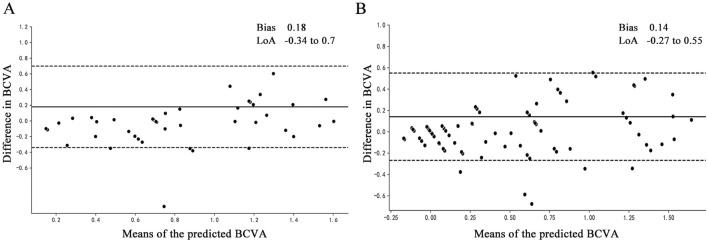
Bland‒Altman plots of the actual BCVA (ground truth) and the predicted BCVA. All vision values were provided in logMAR units. The solid lines show the bias, and the dotted lines show LoAs. BCVA, best corrected distance visual acuity; bias, mean deviation of the prediction from the ground truth; LoA, 95% confidence interval for deviation of the prediction from the ground truth. **(A)** The model established with input parameters for amplitude ratio, PT delay rate, poor mean SE and uncorrected visual acuity, and the amplitude and PT at 15′ and 60′spatial frequencies of healthy eyes. **(B)** The model established with input parameters for mean SE, uncorrected visual acuity, amplitude and PT at 15′ and 60′spatial frequencies of all eyes.

Bland‒Altman plots were used to assess the agreement between ground truth and BCVA predictions, as shown in Figure 4. Figure 4A shows the model established with input parameters for amplitude ratio, PT delay rate, poor mean SE and uncorrected visual acuity, and the amplitude and PT at 15′and 60′spatial frequencies of healthy eyes, where the 95% confidence limits of agreement ranged from −0.34 to 0.70 logMAR and the mean bias between prediction and ground truth was 0.18. The model of mean SE, uncorrected visual acuity, amplitude and PT at 15′and 60′spatial frequencies of all eyes is shown in Figure 4B, with a bias of 0.14% and 95% confidence limits ranging from −0.27 to 0.55 logMAR.

At the same time, data from 20 external eye trauma patients were collected as the validation set, and the results are shown in Table 4.

**TABLE 4:** Experimental results for the validation group.

Model parameters	N	PCC	MAE	RMSE
Both eyes as an individual sample				
Good X1, poor X2 and X3	20	**0.8695**	**0.1951**	**0.2514**
One eye as an individual sample				
X1 and X2	40	**0.9143**	**0.1646**	**0.2274**

X1, The amplitude and peak time at 15′ and 60′ spatial frequencies; X2, mean SE and uncorrected visual acuity; X3, Amplitude ratio and peak time delay rate.

## 4 Discussion

At present, AI is mostly used in ophthalmology to screen, diagnose and predict eye conditions such as glaucoma, AMD, macular edema, DR, and ROP on the basis of OCT, fundus photography, and other sources of data. This technology is also used to evaluate the influence of ametropia and other related factors, such as age, gender, and type of eye injury. In medical diagnosis, DL is mainly correlated with medical imaging analysis. A study collected 120,656 color fundus images of 3,654 patients and carried out classification and evaluation of AMD based on 5,555 fundus images. On that dataset, a deep learning algorithm revealed a better weighted κ than manual classification and was found to be suitable for groups over 55 years old (Grassmann et al., 2018). In addition, AI has shown potential in the research of corneal topography imaging systems, such as the diagnostics of refractive surgery and refractive surgery, visualizing data and further helping doctors make decisions (Shanthi et al., 2022). Liu et al. were devoted to developing a telemedicine platform based on AI analysis of OCT (OCT-AI), which can screen and diagnose patients with retinal diseases in elderly community residents. This online medical model has high sensitivity and specificity, and its clinical effectiveness and cost-effectiveness need to be evaluated in further research (Liu et al., 2022).

AI has also shown good performance in predicting vision. In a study predicting the vision of patients with AMD at 3 and 12 months, machine learning showed good prediction performance, and the best algorithm was the LASSO protocol (Rohm et al., 2018). Wei et al. had explored five different depth learning algorithms to predict the postoperative BCVA of high myopia after cataract surgery from preoperative macular OCT images, which will help the surgical arrangement of cataract patients (Wei et al., 2021). One study developed a feedforward artificial neural network to predict visual outcomes of patients with ROP by patient birth data, follow-up age, and treatment without using biometric data, and it showed an acceptable prediction ability (Huang et al., 2020). Current research focuses on predicting visual acuity through examination of the structure of the eyeball. For example, using electronic medical record data and OCT images, six machine learning models were used to predict visual acuity 1 month after anti-vascular endothelial growth factor treatment (Zhang et al., 2022). In addition, some studies have trained a deep learning model through OCT to predict the 6-month postoperative visual acuity of patients with idiopathic epiretinal membrane, which shows that constructing a vision prediction model is helpful for selecting surgical regimens (Wen et al., 2023). The multimodal model was trained through clinical information and color fundus photography to predict the visual acuity after cataract surgery, which can help to inform the risk and evaluate the effect of surgery (Yang et al., 2025). Other similar studies have confirmed the role of AI in aiding clinical diagnosis (Lin et al., 2024; Wang et al., 2023). The prediction of healing vision in patients with ocular trauma can be used in many clinical fields, which is of great significance for personalized treatment intervention and clinical practice management.

One of the prognostic indicators of eye injury is the Ocular Trauma Score (OTS), which can predict the VA of the injured eye after treatment, plays an essential role in the follow-up diagnosis and treatment of adult patients and is the gold standard method to evaluate the prognosis of adults and children (Kuhn et al., 2002; Morgan and Kasahara, 2018). The functional information on the integrity of the visual pathway from the retina to the occipital cortex is provided by VEPs. A study found that the detection of PVEP is conducive to the discovery of visual pathway disorders in patients with craniosynostosis and subsequent follow-up. The impact of craniofacial surgical intervention on visual pathway function can be evaluated by the changes in PVEP before and after surgery (Haredy et al., 2019). In addition, PVEP is also used to show the changes in the optic nerve caused by hypoxia in patients with obstructive sleep apnea syndrome (OSAS), and the amplitude of the P100 wave increases under positive airway pressure (PAP) (Batum et al., 2020).

In previous studies (Hao et al., 2024), the analysis of PVEP waveforms in ocular trauma recipients through multivariate regression analysis was helpful to further solve the assessment of BCVA after recovery of ocular trauma with different degrees of vision loss and severity of visual acuity, and was conducive to effectively inferring the actual BCVA of the injured eye when it was difficult to obtain a full match of the examinee. Due to the use of analytical methods, the accuracy of predicting visual acuity may be lacking. In this paper, a new analysis method, Machine Learning Models, is used to study a model that better applies PVEP for vision prediction, which is not affected by the professionalism of physicians’ analysis reports, and improves the objectivity of vision identification.

In this study, we found that the XGBoost algorithm evaluated the numerical BCVA of patients with ocular trauma more effectively than the SVR, BYR and RFG algorithms. In addition, XGBoost has shown high accuracy and good performance in BCVA prediction. It was easier to evaluate the VA according to the examination results because the parameters related to the model included PVEP results, basic ophthalmological examination, and OTS category from the past history of ocular trauma. Our study shows that the XGBoost algorithm was designed to achieve acceptable outcomes in predicting the BCVA of patients with basic ophthalmic examination data without entering controversial data.

The use of the XGBoost model in this paper is contributed to predicting the BCVA that patients will achieve after recovery, and it does so while minimizing the degree of interference from the patient’s subjective perceptions and the clinicians’ experience. With the help of the algorithm, vision can be evaluated more conveniently, quickly and objectively, and this tool facilitates tasks such as diagnosis and the detection of special populations. There are many types of ocular trauma involved in this paper, including contusion, lamellar laceration, rupture, penetrating injury, perforating injury, and intraocular foreign bodies. Patients’ vision may be affected by different degrees and types of injury, making it more challenging to predict VA accurately. The influence of human subjective factors is eliminated by using the machine learning algorithm to the greatest extent. This experiment still is limited by a lack of classification data, such as high myopia and other types of ophthalmic diseases (Holden et al., 2016; Wu et al., 2022).

We divided the output variables of the machine learning models into BCVA values. Notably, our model showed a remarkably good Pearson correlation coefficient and low MAE and RMSE for the prediction of numerical BCVA. This proves that the model we selected can be used as an effective tool to assist doctors or forensic experts in predicting patients’ visual function. When inputting parameters to construct an algorithm, the type of outcomes needs to be determined according to the actual situation. The impact of the patient’s age, gender, eye characteristics, and self-reported visual outcomes were eliminated using binocular data from the same individual. If the quantity of patient data is large enough, monocular features could be used. However, it should be noted that this method needs the support of more extensive target population data.

The study was limited by its retrospective design, small sample size, and mixed classification of ocular trauma. In further research, more evaluation indicators and more data need to be included to build a more accurate algorithm. The task of predicting vision remains to be explored in future studies on a larger scale.

## 5 Conclusion

In conclusion, the prediction of VA using PVEP data and individual ophthalmic examination results is a valuable step toward a prognostic decision support system for vision. The system could be used to assist clinicians and forensic experts in predicting visual outcomes for patients with eye injuries.

## Data Availability

The datasets presented in this article are not readily available because The datasets generated and/or analyzed during the current study are not publicly available due to the nature of this research, participants of this study did not agree for their data to be shared publicly. Requests to access the datasets should be directed to WX,xiawt@ssfjd.cn.
